# Characterization of small genomic regions of the hepatitis B virus should be performed with more caution

**DOI:** 10.1186/s12985-018-1100-x

**Published:** 2018-12-10

**Authors:** Lei Jia, Fengyu Hu, Hanping Li, Lin Li, Xiaoping Tang, Yongjian Liu, Haohui Deng, Jingwan Han, Jingyun Li, Weiping Cai

**Affiliations:** 1 Guangzhou Eighth People’s Hospital, Guangzhou Medical University, Guangzhou, 510060 Guangdong China; 2 Department of AIDS Research, State Key Laboratory of Pathogen and Biosecurity, Beijing Institute of Microbiology and Epidemiology, Beijing, 100071 China

**Keywords:** Hepatitis B virus, Genotypic recombination, RDP4, JpHMM

## Abstract

**Background:**

Hepatitis B virus is a hepatotropic DNA virus that reproduces via an RNA intermediate. It can lead to an increased risk of serious liver diseases such as hepatocellular carcinoma and is a serious threat to public health. Currently, the HBV are designated based on greater than 8% nucleotide variation along the whole genome. The recombination of HBV is very common, a large majority of which are recombinants between 2 genotypes. The current work aims to characterize a suspected recombinant involving 3 genotypes.

**Methods:**

Fifty-seven HBV full-genome sequences were obtained from 57 patients co-infected with HBV and HIV-1 by amplification coupled with sequencing. JpHMM and RDP4 were used to perform recombination analysis respectively. The recombination results of a suspected 3-genotypic recombinant were further confirmed by both maximum likelihood phylogenetic tree and Mrbayes tree.

**Results:**

JpHMM recombination analysis clearly indicated one 3-genotypic HBV recombinant composing of B/C/D. The genotype assignments are supported by significant posterior probabilities. The subsequent phylogenetic analysis of sub-regions derived from inferred breakpoints led to a disagreement on the assignment of D segment. Investigating the conflict, further exploration by RDP4 and phylogenies revealed that the jpHMM-derived 3-genotypic recombinant is actually a B/C genotypic recombinant with C fragment spanning 1899 to 2295 (jpHMM) or 1821 to 2199 (RDP4).

**Conclusions:**

The whole analysis indicated that (i) determination of small genomic regions should be performed with more caution, (ii) combinations of various recombination detection approaches conduce to obtain impartial results, and (iii) a unified system of nomenclature of HBV genotypes is necessary.

**Electronic supplementary material:**

The online version of this article (10.1186/s12985-018-1100-x) contains supplementary material, which is available to authorized users.

## Background

Hepatitis B virus (HBV) is a hepatotropic virus with a circular, partial double-stranded DNA genome. It reproduces via an RNA intermediate. HBV virions attack via an entry receptor sodium taurocholate cotransporting polypeptide (NTCP) and infect the liver cells [45]. Chronic HBV infection can lead to an increased risk of severe liver disease, e.g., liver fibrosis, cirrhosis, and hepatocellular carcinoma (HCC) [9, 35, 37]. In this way, it poses a serious threat to public health. HBV has a global distribution with more than 350 million chronic human carriers among whom one third live in China (Hayer et al.; [18]).

Genomic research into HBV was initiated by Galibert in 1979 [8]. In 1988, classification of HBV genome began, based on an 8% sequence difference cutoff over the entire genome [27]. Four genotypes A, B, C, and D were first identified. Under the consideration of the criterion, 4 more genotypes were found [3, 24–26, 32, 40]. Currently, the HBV was designated A to H. Besides, there are two putative genotypes I-J. Based on these genotypes, thousands of HBV complete genomes were recruited in public repositories such as GenBank [4]. Many are inter-genotype recombinant forms due to co-circulation of multiple genotypes in a region. With the development of sequencing and recombination detection techniques, increasing numbers of inter-genotype recombinants have been revealed [1, 2, 6, 7, 19, 44]. Even though the details of genetic recombination of HBV are not fully clear, they may benefit from the proposed intertwinement model for HIV-1 recombination during reverse transcription [15]. The current work aims to characterize a suspected recombinant involving 3 genotypes.

## Materials and methods

### Determination of HBV complete nucleotide sequences

Here, 57 plasma samples were collected from 57 patients co-infected by HBV and HIV-1 in Guangdong Province from 2007 to 2011. The plasma samples were stored at − 80 °C until use. HBV DNA was extracted from plasma samples with the QIAamp DNA Mini Kit (Qiagen). The DNA sample served as a template for HBV DNA amplification by nest-PCR with PrimerStar PCR kit (Takara). Two fragments which could cover full-length of HBV genome were amplified, including long fragment (L fragment, from 1848 to 1807, 3174 bp) and short fragment (S fragment, from 1603 to 2327, 725 bp). All primers were listed in Table 1. The nest-PCR protocol of L fragment was performed as following: 95 °C 2 min, 95 °C 30s, 60 °C 30s, 72 °C 3 min 20s, 30 cycles, in the 1st round of nest-PCR, and then 95 °C 2 min, 95 °C 30s, 60 °C 30s, 72 °C 3 min 20s, 35 cycles, in the 2nd round of nest-PCR. The nest-PCR protocol of S fragment was performed as following: 95 °C 2 min, 95 °C 30s, 60 °C 30s, 72 °C 30s, 30 cycles, in the 1st round of nest-PCR, and then 95 °C 2 min, 95 °C 30s, 60 °C 30s, 72 °C 30s, 35 cycles, in the 2nd round of nest-PCR.

**Table 1:** Primer information for PCR

Primers for PCR		
Primer name	Primer sequence(5′-3′)	Position in HBV genome
HBV-L-F1	GTTCATGTCCWACTGTTCAAGCCTCCAAG	1848–1876
HBV-L-R1	GGTGMRCAGACCAATTTATGCCTACAGCC	1779–1807
HBV-L-F2	CTCCAAGCTGTGCCTTGGGTGG	1870–1891
HBV-L-R2	CAGACCAATTTATGCCTACAGCCTCC	1776–1801
HBV-S-F1	GTCGCATGGARACCACCGTGAA	1603–1624
HBV-S-R1	CCGGAAGTGTTGATAAGATAGGGGCA	2308–2333
HBV-S-F2	GGTCTTRCATAAGAGGACTCTTGGACT	1646–1672
HBV-S-R2	GTGTTGATAAGATAGGGGCATTTGGTGGTCT	2297–2327

PCR products were purified and then sequenced directly. Among these 57 complete sequences, initial jumping profile hidden Markov model (jpHMM) analysis indicated one sequence from a 33-year-old woman in 2016, is a novel B/C/D intergenotype recombinant. It is the first 3-genotypes recombinant discovered in China. Multiple recombination and phylogenetic analyses were performed to confirm this. The HBV genomic DNA is 3215 base pairs in length. It was submitted to GenBank (GenBank accession number: KY417926).The other 56 nucleotide sequences reported in this study were deposited in GenBank under accession numbers MG571321–MG571376.

### Recombination detection

The usual performance of recombination detection on circular viruses is that the circular genomes are first manually linearized and then a linear model is used [6, 23, 32, 38, 41]. A major defect of this strategy is that dependencies between nucleotides at the 5′ and 3′ end of a sequence cannot be modeled [32]. It is known that when linear sequences of an alignment are analyzed as though they were circular and some recombination is detected, it becomes possible to detect a strong recombination hotspot spanning the beginning and end of the analyzed sequences [20, 21]. In contrast, when circular genomes are analyzed as though they were linear, recombination breakpoints occurring closely to the 5′ or 3′ ends of the linearized sequence may be overlooked [32].

The detailed recombination analysis is performed as previously described [14–17, 43, 46]. Both the jpHMM and recombination detection program (RDP4) used in the current work take the circularity of the HBV genomic sequences into consideration and can facilitate accurate predictions of recombination breakpoints even close to the 5′or 3′end of the linearized sequence [21, 32]. As demonstrated in a previous study [16], jpHMM and RDP can easily produce highly accurate and impartial recombination data.

JpHMM was first used to perform recombination analysis. This tool is very intelligent and can produce a genome mosaic map with position numbers given either in the original sequence or relative to the HBV reference genome AM282986. The recombination prediction in jpHMM is based on a precalculated multiple sequence alignment of the major HBV reference genotypes, and the evaluation of its prediction accuracy showed that it to be more accurate than the competing methods used for phylogenetic breakpoint detection [32, 33, 46].

To confirm the data obtained by jpHMM, another recombination analysis tool, RDP4, was used for further analysis. RDP4 is a software package suitable for statistical identification and characterization of recombination events in nucleotide sequences [20, 21]. RDP4 is also very intelligent and utilizes a range of non-parametric recombination detection methods simultaneously: RDP, GENECONV [28], BOOTSCAN [22, 31], MAXCHI [29, 39], CHIMAERA [29], SISCAN [10], 3SEQ [5], and LARD [13], which can greatly increase sensitivity. Here, the sequences were set to *linear*. The highest acceptable *P*-value was set to 0.05. The other parameters are default RDP4 settings. To ensure reliability, the HBV sequence were considered recombinant when the recombination signal was supported by at least 4 methods with *P*-values of ≤0.05 after Bonferroni correction for multiple comparisons implemented in RDP4 [17, 20, 36]. The breakpoint positions inferred were manually checked using recombination signal analysis implemented in RDP4. Details regarding the methods and algorithms of the recombination analysis tools used here are given in the comprehensive list of recombination analysis software maintained by the Robertson Lab (http://www.bioinf.manchester.ac.uk/robertson/recombination/programs.shtml).

### Phylogenetic analysis

To further confirm the results of the recombination, maximum likelihood phylogenetic trees (ML) were constructed based on the inferred breakpoint locations using the PhyML 3.0 implemented in RDP4 [11]. Automatic model selection with PhyML3.0 was used to find the best-fitting model of nucleotide substitution. Tree topologies were searched using NNI and SPR procedure. The confidence of each node in phylogenetic trees was determined using the bootstrap test with 1000 bootstrap replicates. The final ML trees were visualized using Mega 6 [42].

### Reference sequence selection

The clear and unified definition of genotype representatives is important to both recombination analysis by RDP4 and subsequent confirmation by phylogenetic analysis. All reference sequences are selected based on previous works and HBVdb [6, 12]. These sequences were all further verified via jpHMM to ensure the consistency. Inconsistent values were all removed from the references alignment.

## Results

### Recombination screening of all 57 full-length sequences

Here, 7.02% of the sequences (4 out of 57) were identified as genotypes C by jpHMM. 91.23% of the sequences (52 out of 57) were identified as BC intergenotypic recombinants, i.e. genotype Ba. The 52 BC recombinants all had a fragment of genotype C over the precore region plus the core gene. This type of HBV has been previously discovered and is very common in southern China. Specifically, one sequence is typically identified as a 3-intergenotype recombinant comprising the B, C, and D genotypes. Although there are multiple genotypes co-circulating in China, such as B, C and D, a 3-genotype recombinant has never been found.

### JpHMM analysis revealed a HBV recombinant between 3 genotypes

**Fig. 1:**
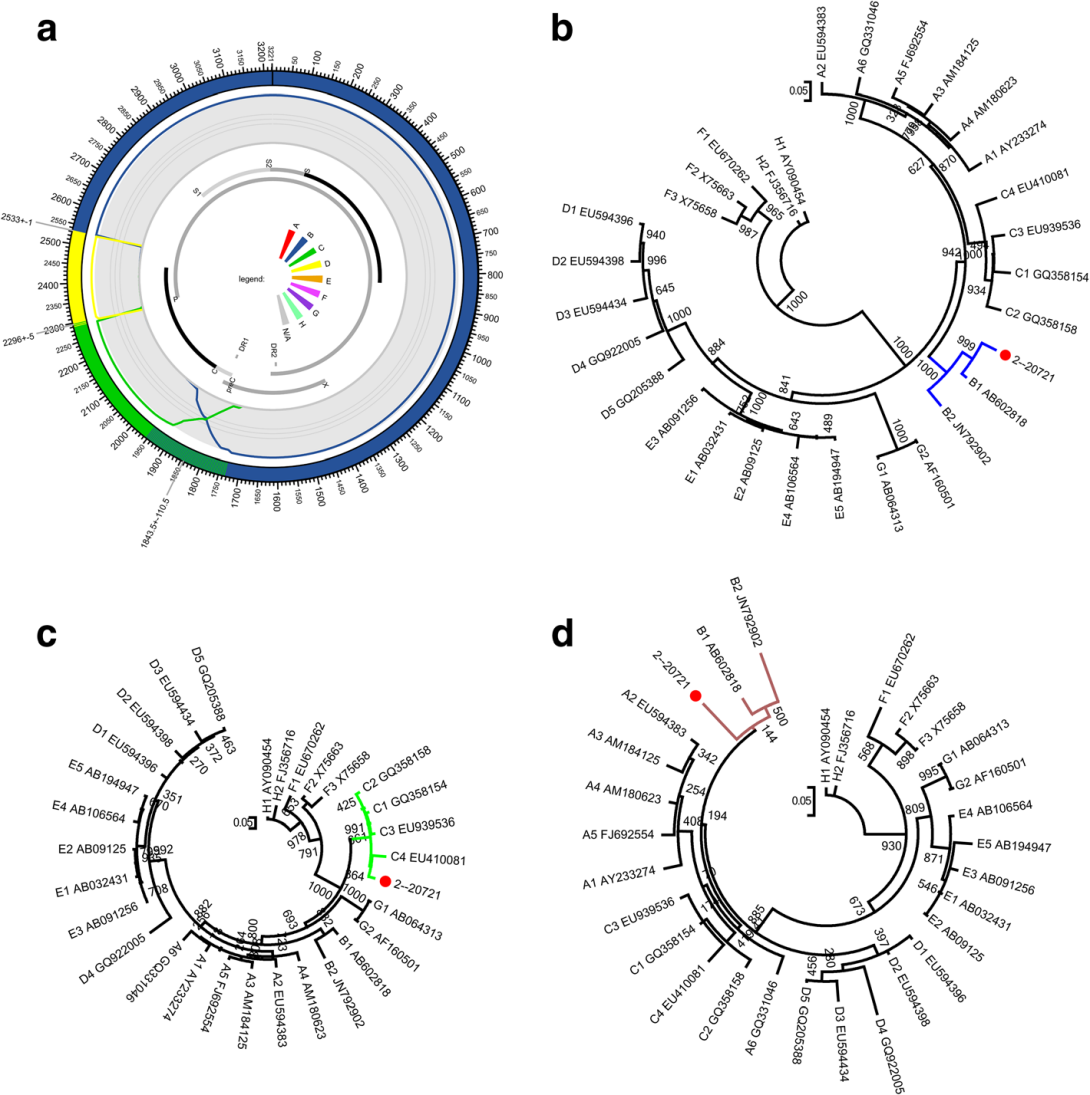
The jpHMM-derived mosaic structure and confirmation by sub-region phylogenetic trees. **a** The jpHMM-derived mosaic structure. The predicted genotype recombination is represented in the outer ring. Posterior probabilities of the genotypes at each sequence position were calculated using jpHMM and are plotted in the second inner ring. P, C, S, and X indicate polymerase, core, surface, and X genes. **b** ML (GTR + I + G4) phylogenetic tree of regions derived from major parent (1–1898 and 2526–3215 of the strain). **c** ML (GTR + I + G4) phylogenetic tree of the region spanning 1899–2295 of the strain. **d** ML (GTR + I + G4) phylogenetic tree of the region spanning 2296–2525 of the strain. Phylogenetic trees were constructed using the PhyML 3.0 implemented in RDP4. The reliability of the phylogenetic tree analysis was assessed by bootstrap resampling with 1000 replicates. Genotypes and GenBank accession numbers are indicated. Values at the nodes indicate the bootstrap numbers by which the cluster was supported. Branch lengths are drawn to scale

The jpHMM analysis of the strain obtained in the current work (GenBank accession number: KY417926) indicated clearly that it is a recombinant related to B, C, and D genotypes. The breakpoints clearly located at 1899, 2296, and 2526, respectively in the original sequence. The genotype assignments are supported by significant posterior probabilities (Fig. 1a, Table 2). Recombination of HBV between 2 genotypes is very common. However, recombinants involving more than 2 genotypes are rare [2]. To date, “genotype” I has been shown to be related to 3 genotypes: A/C/G [44].

**Table 2:** Positions of the jpHMM-derived breakpoints in the original sequence

Fragment Start Position	Uncertainty Region Start - End	Breakpoint Interval Start - End	Fragment End Position	Fragment Genotype
1	–	1733–1954	1898	B
1899	–	2291–2301	2295	C
2296	–	–	2525	D
2526	–	–	3215	B

In order to confirm the results of jpHMM, the 3 sub-regions delimited by the jpHMM-derived breakpoints in the strain were used to construct an ML tree with genotype references, respectively. The computed GTR + I + G4 model by PhyML3.0 was suitable for all trees. The tree of the B genotype region (spanning 1–1898, 2526–3215) clearly showed the clustering of the fragment with the B references (Fig. 1b). The tree of the region spanning 1899 to 2295 clearly showed the clustering of the fragment with the C references (Fig. 1c). Both these results are consistent with the jpHMM results. However, an apparent conflict became visible in the determination of the genotype assignment of the region spanning 2296 to 2525 (230 bases). Despite the low bootstrap values, the tree clearly showed that the fragment, unexpectedly, did not cluster with D references but rather with the B references (Fig. 1d).The topology of the tree was further confirmed by another round of phylogenetic analysis using the MrBayes tool implemented in RDP4 based on Bayesian inference [30].As shown in Additional file 1: Figure S1, the Bayesian tree showed the same results with respect to the assortment of D fragment.

### RDP4 analysis indicates that the jpHMM-derived 3 genotypic recombinant is actually a 2 genotypic recombinant

**Fig. 2:**
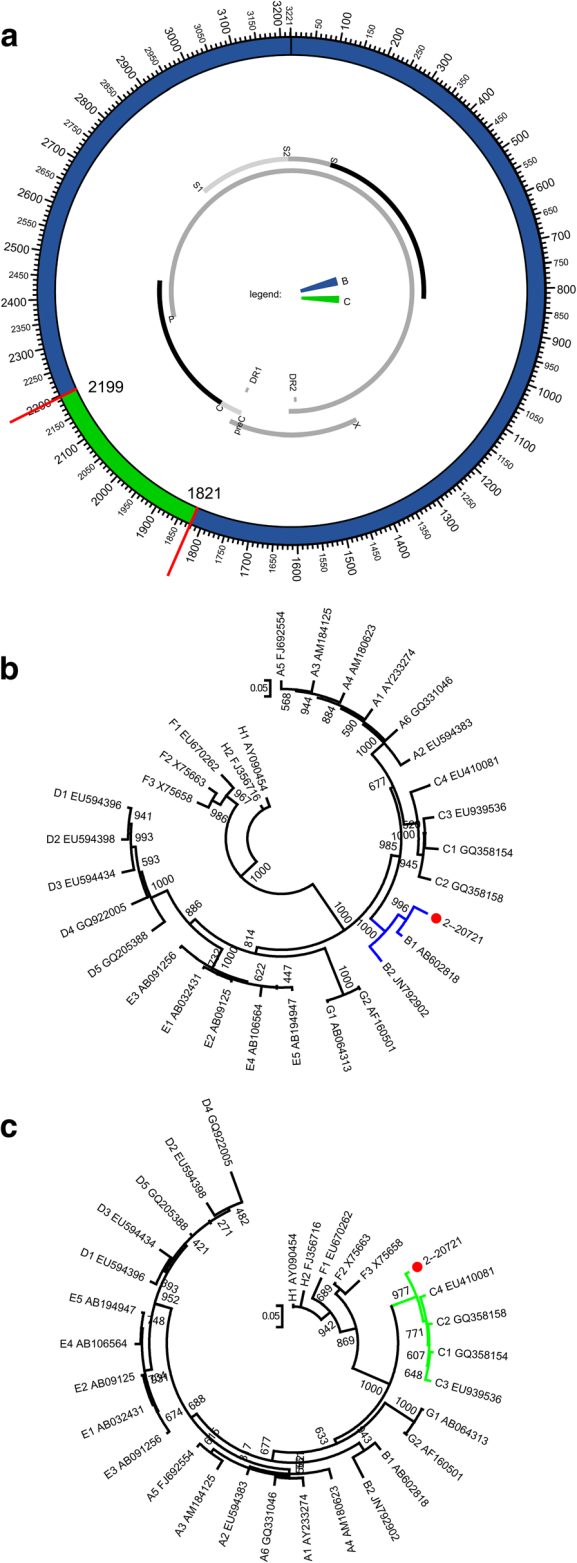
The RDP-derived mosaic structure and confirmation by sub-region phylogenetic trees. **a** The RDP-derived mosaic structure. The predicted genotype recombination is represented in the ring. The predicted breakpoints are displayed. P, C, S, and X indicate polymerase, core, surface, and X genes. **b** ML (GTR + I + G4) phylogenetic tree of regions derived from major parent (1–1820 and 2200–3215 of the strain). **c** ML (GTR + G4) phylogenetic tree of regions derived from minor parent (1821–2199 of the strain).Phylogenetic trees were constructed using the PhyML 3.0 implemented in RDP4 . The reliability of the phylogenetic tree analysis was assessed by bootstrap resampling with 1000 replicates. Genotypes and GenBank accession numbers are indicated. Values at the nodes indicate the bootstrap numbers by which the cluster was supported. Branch lengths are drawn to scale

To investigate this discrepancy, another recombination analysis tool, RDP4, was used to further explore the mosaic structure of the strain. The unique tool simultaneously utilizes a range of non-parametric recombination detection methods and thus has increased sensitivity and reliability. Significantly different from the result from jpHMM, RDP4 analysis clearly indicates that the strain is a B\C recombinant, excluding any other genotypes, with the breakpoints located at 1821 and 2199 in the original sequence (Fig. 2a, Table 3). This recombination event is supported by all 8 recombination detection methods (*P*-values are listed in Additional file 1: Table S1). Subsequent confirmations of genotype assignment by phylogeny are consistent with the RDP4 results. The major parent clustered with B references and 1000 replicates supported (Fig. 2b). The minor parent clustered with C references and 977 replicates supported (Fig. 2c). Thus, 2 major recombination tools have been used, variously displaying the recombination pattern of the HBV strain. Given that 2 of the 3 methods (jpHMM, RDP4, and the subsequent phylogenies) indicated a similar recombination event, the strain identified in the current work is assigned to a B/C recombinant, i.e. Ba, with C fragment spanning 1899 to 2295 (jpHMM) or 1821 to 2199 (RDP4). The breakpoint positions relative to reference genome AM282986 numbering are given in Additional file 1: Table S2–S3. All the other 56 stains were also further confirmed by RDP4 and the results are accordance with those from jpHMM.

**Table 3:** Positions of the RDP4-derived breakpoints in the original sequence

Fragment Start Position	Uncertainty Region Start - End	Breakpoint Interval Start - End	Fragment End Position	Fragment Genotype
1	–		1820	B
1821	–		2199	C
2200	–	–	3215	B

## Discussion

JpHMM significantly indicate a typical recombinant related to 3 genotypes: B, C, and D. When the mosaic structure was confirmed as usual by phylogenetic analysis based on jpHMM-derived sub-regions, a conflict arose regarding the genotype assignment of the D fragment. The sub-region tree shows that the segment clusters with B references (Fig. 1d). To further validate the unexpected disagreement, RDP was used to re-characterize the strain. Results indicated a confessedly B/C recombinant with C region spanning 1821 to 2199. Subsequent phylogenies confirmed this characterization (Fig. 2). In this way, the strain was finally identified as a B/C recombinant with a C fragment spanning 1899 to 2295 (jpHMM) or 1821 to 2199 (RDP4), i.e. Ba, due to 2 of the 3 performance (jpHMM, RDP4, and the phylogenies) indicating a similar recombination event.

The jpHMM-derived genotype D region includes 230 bases. In HIV-1, Leitner et al. pointed out such small regions of about 200 bases or fewer include too little sequence information to produce reliable phylogenetic trees (http://www.hiv.lanl.gov/content/sequence/HIV/REVIEWS/RefSeqs2005/RefSeqs05.html). The reanalysis of the recombination of some HIV-1 circulating recombination forms found that many previously identified small fragments with fewer than 200 bases cannot be detected using most methods [16]. HBV showed a lower variation rate than HIV. In exactly the same way, characterization of small fragments in HBV must be performed with great caution. It is here suggested that the involvement of multiple recombination detection programs and multiple approaches may facilitate the production of impartial results. In the future, with improvement in the understanding of recombination mechanisms and in the recombination detection techniques, much more optimized resolutions of identifying small fragments may be developed.

Well-defined genotype references are another critical factor for detecting recombination in HBV genome sequence alignment. Unlike the representative sequences shown in Table 4 applied to RDP and phylogenies analysis, each genotype used in jpHMM analysis is modeled as a profile Hidden Markov Model (HMM) which is built based on adequate sampling of the genotype [32–34, 46]. All profile models are connected by empirical probabilities. Despite all references for both RDP4 and phylogenies have been validated by jpHMM, differences in the strategies by which references are constructed may also be one cause of the conflicting results.

**Table 4:** Details of the selected references

Genotype	References	Accession number
A	A_1	AY233274
	A_2	EU594383
	A_3	AM184125
	A_4	AM180623
	A_5	FJ692554
	A_6	GQ331046
B	B_1	AB602818
	B_2	JN792902
C	C_1	GQ358154
	C_2	GQ358158
	C_3	EU939536
	C_4	EU410081
D	D_1	EU594396
	D_2	EU594398
	D_3	EU594434
	D_4	GQ922005
	D_5	GQ205388
E	E_1	AB032431
	E_2	AB09125
	E_3	AB091256
	E_4	AB106564
	E_5	AB194947
F	F_1	EU670262
	F_2	X75663
	F_3	X75658
G	G_1	AB064313
	G_2	AF160501
H	H_1	AY090454
	H_2	FJ356716

It is here noted that some representatives of HBV are not unified. The genotype references adopted in the current work originated from 2 resources. One is a previous publication by Chen et al [6]. The other is the HBV database [12]. However, when validated by jpHMM, the surprising thing is that 4 of 6 B references (accession number: AF282918, GQ358136, AB368295, and GQ924624) provided by Chen et al. have been determined to be B/C recombinants [6]. Both references of genotype B (accession number: AB219428 and D00331) provided by the HBV database have been determined to be B/C recombinants [12]. One of 5 C genotypes (accession number: GQ377630) provided by Chen et al. have been determined to be B/C recombinants (Additional file 1: Figure S2) [6]. Although these problematic representatives were all excluded during the analysis, they clearly suggest a non-unified definition of HBV genotypes. Pure B without recombination was classified as Bj (j indicating for Japan) and Ba (a indicating Asia) was identified as recombinant with genotype C over the precore region plus the core gene [41]. Obviously it is not appropriate to list these recombinant strains as representatives of pure B genotype. In summary, a unified system of nomenclature of HBV genotypes is significantly necessary.

## Conclusions

Impartial recombination analysis critically depends on 2 factors. One is effective and ingenious recombination detection tool, and the other is a clear definition of pure genotypes. However, in the current work, identification and characterization of a seemingly certain recombinant involving 3 genotypes indicated that (i) determination of small genomic regions should be performed with more caution, (ii) combinations of various method of recombination detection conducive to reaching unbiased results, and (iii) a unified system of nomenclature of HBV genotypes is required.

## Additional file

Additional file 1: **Table S1.** Details of the recombination results of the strain by 8 analysis methods implemented in RDP4. **Table S2.** JpHMM-derived breakpoints position based on reference genome AM282986 numbering. **Table S3.** RDP4-derived breakpoints position based on reference genome AM282986 numbering. **Figure S1.** The phylogenetic analysis of the assortment of D fragment using MrBayes tool implemented in RDP4 based on Bayesian inference. All 6 substitution types can be unequally likely. Auto-correlated gamma-distributed variation is selected. The number of rate categories is 4. The number of generations is 1,000,000,000. The sampling frequency is 100. The number of chains is 4. The temperature is 0.2. the swap frequency is 1. The swap number is 1. **Figure S2.** JpHMM-derived recombination pattern of 7 references. (a-f) Mosaic structures of genotype B (accession number: AF282918, GQ358136, AB368295, GQ924624, AB219428, and D00331). (g) Mosaic structure of genotype C (accession number: GQ377630). (PDF 489 kb)

## Abbreviations

HBV: Hepatitis B virus
HCC: Hepatocellular carcinoma
jpHMM: Jumping profile hidden Markov model
ML: Maximum likelihood
NTCP: Sodium taurocholate cotransporting polypeptide
RDP: Recombination detection program
