# Supplementary material for: Characterization of small genomic regions of the hepatitis B virus should be performed with more caution
Source: Virol J. 2018 Dec 10;15:188. doi: 10.1186/s12985-018-1100-x (PMC6288937; doi:10.1186/s12985-018-1100-x)
Supplement: Supplementary file 1 — Table S1. Details of the recombination results of the strain by 8 analysis methods implemented in RDP4. Table S2. JpHMM-derived breakpoints position based on reference genome AM282986 numbering. Table S3. RDP4-derived breakpoints position based on reference genome AM282986 numbering. Figure S1. The phylogenetic analysis of the assortment of D fragment using MrBayes tool implemented in RDP4 based on Bayesian inference. All 6 substitution types can be unequally likely. Auto-correlated gamma-distributed variation is selected. The number of rate categories is 4. The number of generations is 1,000,000,000. The sampling frequency is 100. The number of chains is 4. The temperature is 0.2. the swap frequency is 1. The swap number is 1. Figure S2. JpHMM-derived recombination pattern of 7 references. (a-f) Mosaic structures of genotype B (accession number: AF282918, GQ358136, AB368295, GQ924624, AB219428, and D00331). (g) Mosaic structure of genotype C (accession number: GQ377630). (PDF 489 kb) [file 12985_2018_1100_MOESM1_ESM.pdf]

Table S1. Details of the recombination results of the strain by 8 analysis methods implemented in RDP4.

| Recombination methods | RDP | GENECONV | Bootscan | Maxchi | Chimaera | SiSscan | LARD | 3Seq |
|---|---|---|---|---|---|---|---|---|
| *p*-value | 1.76E-13 | 3.28E-10 | 2.47E-14 | 3.13E-04 | 1.60E-04 | 9.01E-08 | 3.53E-13 | 1.62E-12 |

Table S2. JpHMM-derived breakpoints position based on reference genome AM282986 numbering.

| | | | | |
|---|---|---|---|---|
| **1** | - | 1733 - 1954 | **1898** | **B** |
| **1899** | - | 2291 - 2301 | **2295** | **C** |
| **2296** | - | - | **2531** | **D** |
| **2532** | - | - | **3221** | **B** |

Table S3. RDP4-derived breakpoints position based on reference genome AM282986 numbering.

| Fragment Start Position | Uncertainty Region Start - End | Breakpoint Interval Start - End | Fragment End Position | Fragment Genotype |
|---|---|---|---|---|
| **1** | - | | **1820** | **B** |
| **1821** | - | | **2199** | **C** |
| **2200** | - | - | **3215** | **B** |

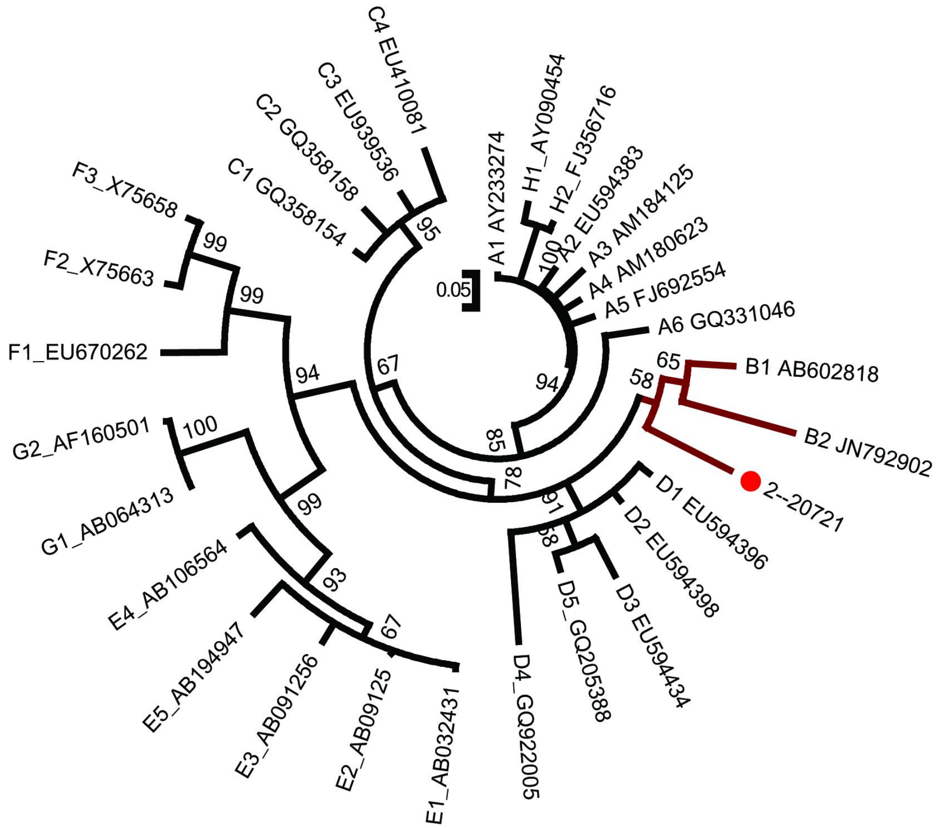


Figure S1. The phylogenetic analysis of the assortment of D fragment using MrBayes tool implemented in RDP4 based on Bayesian inference. All 6 substitution types can be unequally likely. Auto-correlated gamma-distributed variation is selected. The number of rate categories is 4. The number of generations is 1000000000. The sampling frequency is 100. The number of chains is 4. The temperature is 0.2. the swap frequency is 1. The swap number is 1.

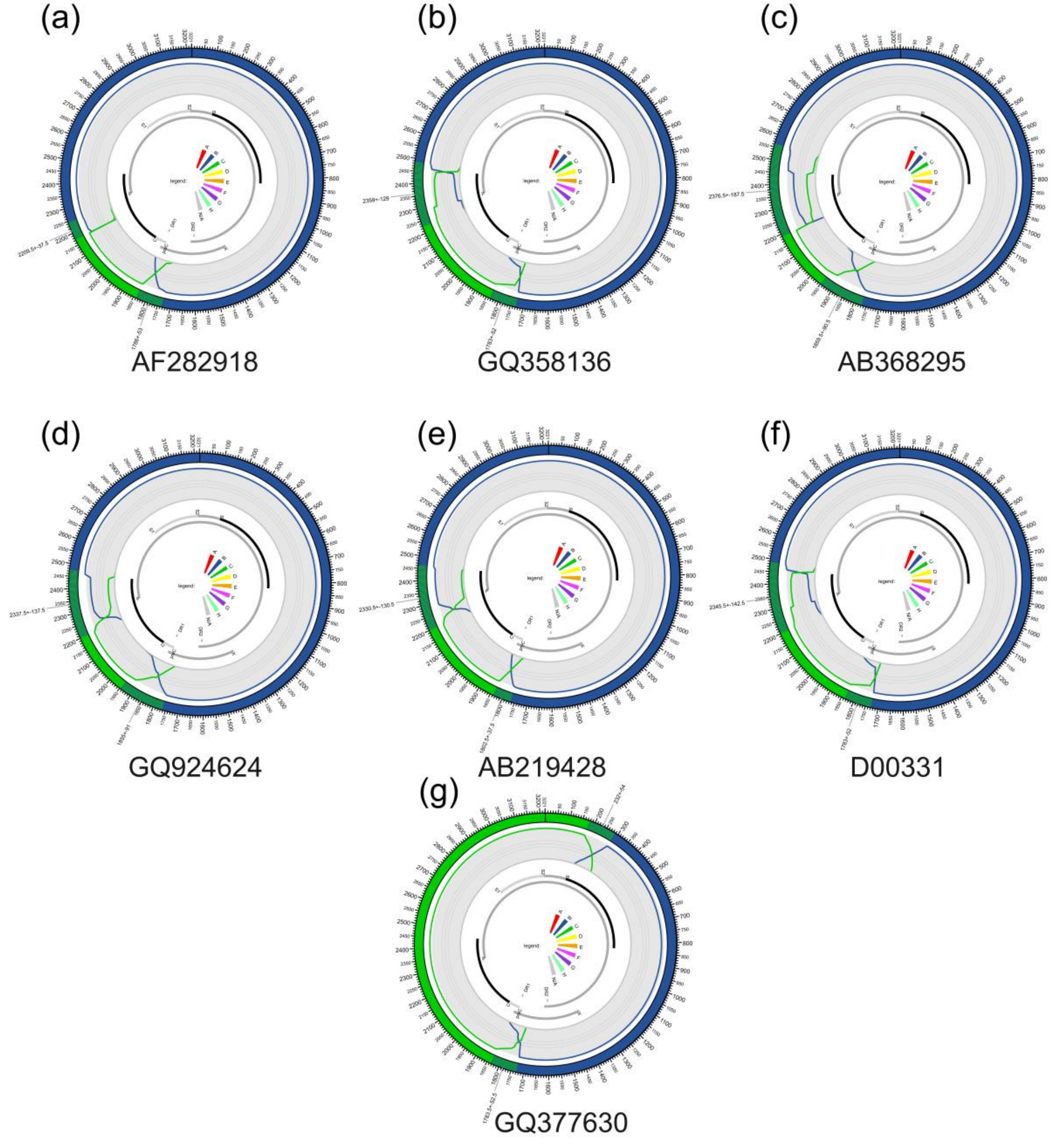
(a)
AF282918
(b)
GQ358136
(c)
AB368295
(d)
GQ924624
(e)
AB219428
(f)
D00331
(g)
GQ377630

Figure S2. JpHMM-derived recombination pattern of 7 references. (a-f) Mosaic structures of genotype B (accession number: AF282918, GQ358136, AB368295, GQ924624, AB219428, and D00331). (g) Mosaic structure of genotype C (accession number: GQ377630).
